# Comparison of self-reported health & healthcare utilisation between asylum seekers and refugees: an observational study

**DOI:** 10.1186/1471-2458-9-214

**Published:** 2009-06-30

**Authors:** Magzoub Toar, Kirsty K O'Brien, Tom Fahey

**Affiliations:** 1 Department of General Practice, Royal College of Surgeons in Ireland Medical School, Beaux Lane House, Mercer Street Lower, Dublin 2, Ireland; 2 HRB Centre for Primary Care Research, Department of General Practice, Royal College of Surgeons in Ireland Medical School, Beaux Lane House, Mercer Street Lower, Dublin 2, Ireland

## Abstract

**Background:**

Adult refugees and asylum seekers living in Western countries experience a high prevalence of mental health problems, especially post traumatic stress disorder (PTSD), depression and anxiety. This study compares and contrasts the prevalence of health problems, and potential risk factors as well as the utilisation of health services by asylum seekers and refugees in the Irish context.

**Methods:**

Cross sectional study using validated self reported health status questionnaires of adult asylum seekers (n = 60) and refugees (n = 28) from 30 countries, living in Ireland. Outcome measures included: general health status (SF-36), presence of PTSD symptoms and anxiety/depression symptoms. Data on chronic conditions and pre or post migration stressors are also reported. The two groups are compared for utilisation of the health care system and the use of over the counter medications.

**Results:**

Asylum seekers were significantly more likely than refugees to report symptoms of PTSD (OR 6.3, 95% CI: 2.2–17.9) and depression/anxiety (OR 5.8, 95% CI: 2.2–15.4), while no significant difference was found in self-reported general health. When adjusted by multivariable regression, the presence of more than one chronic disease (OR 4.0, 95%CI: 1.3–12.7; OR 3.4, 95% CI: 1.2–10.1), high levels of pre migration stressors (OR 3.6, 95% CI: 1.1–11.9; OR 3.3, 95% CI: 1.0–10.4) or post migration stressors (OR 17.3, 95% CI: 4.9–60.8; OR 3.9, 95% CI: 1.2–12.3) were independent predictors of self reported PTSD or depression/anxiety symptoms respectively, however, residence status was no longer significantly associated with PTSD or depression/anxiety. Residence status may act as a marker for other explanatory variables; our results show it has a strong relationship with post migration stressors (χ^2 ^= 19.74, df = 1, P < 0.001).

In terms of health care utilisation, asylum seekers use GP services more often than refugees, while no significant difference was found between these groups for use of dentists, medication, hospitalisation or mental health services.

**Conclusion:**

Asylum seekers have a higher level of self reported PTSD and depression/anxiety symptoms compared to refugees. However, residence status appears to act as a marker for post migration stressors. Compared to refugees, asylum seekers utilise GP services more often, but not mental health services.

## Background

Population based studies on adult refugees and asylum seekers living in Western countries report a high prevalence of mental health problems, particularly symptoms suggestive of post traumatic stress disorder (PTSD), depression and anxiety [1]. Most studies are based on relatively small samples of patients and include people from a single country of origin [1]. Furthermore, the majority of studies focus either on refugees or on asylum seekers alone, without contrasting their health status and different health experiences.

Compared to other Western countries Ireland received a relatively small number of refugees until the early 1990s. Since this time the annual number of people seeking refugee status has risen substantially to a peak of over 11,000 applications in 2002 [2]. This increase has led to concerns about the health and quality of life of asylum seekers and refugees in Ireland.

On an annual basis, approximately 9% of asylum seekers are granted refugee status in Ireland (2002–2007 figures [2]). While their application is being processed asylum seekers in Ireland are required to live in direct provision centres (shared hostel-type accommodation), where all their meals are provided and they receive an allowance of approximately €19 per week [3]. During this time they are legally prohibited from working, however, they are entitled to adult literacy and English language courses. Legal Aid and healthcare are means tested; anyone who qualifies for a medical card (asylum seeker or refugee) is entitled to free healthcare, including access to a general practitioner, prescribed medications and psychology services [3].

Ireland has also been a member of the UNHCR Refugee Resettlement Program since 1998 and admits approximately 140 refugees per year under this scheme (2005–2007 figures [3]). These programme based or 'offshore refugees' do not undergo the asylum process in Ireland as they are granted refugee status prior to their arrival in Ireland and have therefore not been included in this study.

Refugees and asylum seekers differ in a number of ways that may affect health status and the utilisation of health care services; for example, in terms of living arrangements, the direct provision centres and the inability to seek work may negatively impact on the health and wellbeing of asylum seekers.

The process of seeking refugee status can take a considerable amount of time in Ireland, with 32% of asylum seekers residing in direct-provision centres for over two years [4]. The length of asylum procedure has been shown to be independently associated with an increased risk of psychiatric disorders [5]. Previous studies report a broad range in prevalence of PTSD from 4% to 70%, and a similarly wide range of prevalence concerning depression (3% to 88%) and anxiety (2% to 80%) in refugees and asylum seekers [1]. The subsequent impact of poor mental health and its influence on health seeking behaviour and health care utilisation has not been previously addressed in the Irish context.

The aim of this study is to provide and compare epidemiological data on the self-reported health status of refugees and asylum seekers using validated instruments [5,6]. We will explore any differences in health between these two groups to discover if residence status is an independent predictor of health outcomes or if it acts as a marker for other risk factors. Data will also be collected on subsequent health care utilisation, allowing a comparison and better understanding of health and health care utilisation of refugees and asylum seekers.

## Methods

### Design, Setting and Participants

We performed a cross sectional study using validated questionnaires of self-reported health status. The study sample consisted of adult asylum seekers and refugees from thirty countries including Iran (4), Iraq (7), Afghanistan (4), Pakistan (6), Vietnam (1), Bosnia (1), Bhutan (2), Georgia (2), Kazakhstan (1), Kuwait (2), Lebanon (1), Russia (1) and a number of African countries (56). The target population of this study was asylum seekers living in two direct provision centres in Sligo and Leitrim, Ireland and refugees who were living in the same community services area. The questionnaire included questions on physical health, mental health, utilisation of health care services, pre and post migratory traumatic experiences and each person's socio-demographic background.

### Procedures

Participants in this study were sent a letter and consent form, informing them about the study, the questionnaire, confidentiality, and the voluntary nature of participation. Asylum seekers were assured that participation in the study would have no influence on their asylum request. The study protocol was approved by the Research Ethical Committee of Royal College of Surgeons in Ireland.

With permission from the authors, a modified version of a questionnaire developed in the Netherlands was used in this study [6]. The questionnaire was modified by the removal of sections on life-style, acculturation and social support. To minimize the risk of misunderstanding or miscommunication and to address difficulties in relation to translation or clarification on items, one of the researchers (MT) administered the questionnaire to all participants in a face to face interview and used an interpreter when necessary to overcome any language difficulties.

### Sampling and sample size calculation

All asylum seekers and refugees included in this study were 18 years of age or above. As a family tend to have similar values for some of the outcomes studied, only one member per family was randomly selected on the basis of a registration code.

Offshore refugees were excluded from the study as they are granted refugee status prior to arrival in Ireland and have never been through the asylum process.

The sample size was calculated based on the results of a previous study that reported the proportion of asylum seekers and refugees with PTSD symptoms using the Harvard Trauma Questionnaire (HTQ) scoring system [7]. We planned to recruit between 53–66 asylum seekers and 27–33 refugees, assuming a difference in the proportion of patients with PTSD symptoms to be 32.4% (43.4% for asylum seekers and 11% for refugees) [7]. This would give the study a power of between 80–90% at a significance level of 0.05.

The recruitment was carried out over a three month period (February to April 2007). A random sample of asylum seekers (66 people) were selected from the register of the two direct provision centres and asked to participate in the study. For the refugee sample, Community Welfare Officers have a register of the refugees living in their catchment area, and a random sample from this register (33 people) were asked to participate in the study.

### A) Health Status

#### Outcome Measures

We assessed three principal outcomes in relation to health: general health status; symptoms of PTSD; and symptoms of anxiety and depression. For analyses, the health outcome variables were dichotomised as follows.

**1. General health status **– current health status of respondents was measured according to the general health questions on the 36-item Short-Form (SF-36) [8]. The response options ranged from '5 = excellent' to '1 = poor' and were subsequently dichotomised into: 'good' (excellent, very good, good) and 'poor' (fair, poor). Although not originally developed for use in migrant populations, the SF-36 has been translated into numerous languages for use in research. However, it should be noted that its cross cultural validity has been questioned [5].

**2. PTSD status **– part IV of the Harvard Trauma Questionnaire (HTQ) was used [9]. It includes 30 symptoms, of which 16 were derived from the Diagnostic and Statistical Manual of Mental Disorders (DSM-III-R) criteria for PTSD and 14 describe symptoms related to the traumatic events in the refugees' lives. The format of the response options is comparable to that of the HSCL-25 (item 3 below). Individuals with a mean score on the 16 PTSD symptoms and/or on the total list of 30 symptoms = 2.5 were considered to be symptomatic for PTSD. This scale was developed for use in refugee populations and has been used in numerous studies on refugees [1,5]. The 2.5 cut-point was chosen to make our study comparable to others, however, it should be noted that the validity of this cut point has only been demonstrated in outpatient psychiatric patients and not in a community based setting [5].

**3. Anxiety and depression status **– the Hopkins Symptom Check List-25 (HSCL-25) [10,11], was used to measure symptoms of anxiety (10 items) and depression (15 items). Respondents were asked to indicate the extent to which they were bothered by each symptom in the previous week, ranging from '1 = not at all' to '4 = extremely'. Individuals with a mean score for anxiety and/or depression and/or the total list of symptoms > 1.75 were considered to be symptomatic. This instrument wasn't originally designed for use in refugee populations but has been adapted [11], and subsequently used in a number of refugee studies [1,5]. The cut point of 1.75 was chosen so our results would be comparable with other studies. However, the validity of this cut point in community based refugees and asylum seekers of multiple nationalities is unclear.

### Explanatory variables

To explore the influence of potential explanatory variables on general health, PTSD and depression/anxiety, we assessed the following:

**1. Residence status **– refugee or asylum seeker.

**2. Pre migration stressors **– Pre migration stressors (traumas) were assessed with part 1 of the HTQ, which includes 17 events (e.g. lack of food and water, being close to death) with three possible responses (experienced, witnessed, no) [9]. An additional 15 traumatic events (e.g. rocket attacks) were added to the questionnaire by Gerritsen and colleagues based on Amnesty International Annual Reports (1975–2002) [6] as well as items from HTQ part III that focus on traumatic events. This variable was dichotomised into '0 to 7 pre migration traumas' and '≥ 8 pre migration traumas'.

**3. Post migration stressors **– Respondents were asked about possible stressful experiences they have experienced in Ireland. The checklist included 18 problems often reported by refugees in research on post migration stressors (e.g. uncertainty about residence status, loneliness, discrimination and communication problems) [6,12-14]. They were asked to indicate the extent to which any of the items had bothered them in the previous month ('1 = not at all' to '4 = extremely'). A mean score was calculated and dichotomised into < 2.5 and ≥ 2.5 events [15].

**4. Numbers of chronic conditions **– Respondents were asked to indicate for 28 chronic conditions (e.g. severe headache, diabetes) whether or not they had had this condition in the last 12 months. As done elsewhere, this variable was dichotomised into '0 or 1' and '>1' chronic condition [15].

### B) Health care Utilisation

Utilisation of Health Care Services was assessed by recording the following self reported data: (i) frequency of contact with a general practitioner, outpatient medical specialist, nurse or area medical officer in the previous two months; (ii) hospital admissions or contact with mental health service in previous 12 months; (iii) use and type of prescribed and over-the-counter medication in the previous 14 days.

### Analysis of Results

SPSS v.15 was used for analysis. A comparison was made between asylum seekers and refugees using two-tailed Pearson chi-square and Student's t-tests to examine differences in socio-demographic variables and other risk factors. Differences in health outcomes and the use of health care services between the two groups were examined by calculating the odds ratios (ORs), 95% confidence intervals and *P *values using univariable analyses.

We examined a number of potential confounding factors including socio-demographic factors (e.g. gender, age, marital status) as well as period of residence in Ireland and highest level of education completed. These variables were explored in univariable analysis with general health, PTSD and depression/anxiety as the health outcomes.

To identify factors that were independently associated with health outcomes and with use of health care services; multivariable logistic regression analyses was performed by the inclusion of explanatory variables that were significant (P < 0.05) on univariable analysis. Adjusted odds ratios (OR) were calculated using a backwards conditional multivariable logistic regression models to control for the presence of other variables (removal probability limit of P > 0.10).

## Results

### Characteristics of study population

For three months (Feb 2007 to April 2007) a total of 66 asylum seekers were invited to participate in this study, of these 60 asylum seekers agreed to be interviewed (response rate of 91%). A total of 33 refugees living in the community were invited to participate, and 28 were interviewed (response rate of 85%). The respondents were from 30 different countries.

Table 1 compares the characteristics of refugees and asylum seekers. Refugees had a significantly higher level of educational attainment (χ^2^= 8.68, df = 2, P = 0.01), a lower level of post migration stressors (χ^2^= 19.74, df = 1, P < 0.01), and had spent more time in Ireland when compared to asylum seekers. The most common post migration stressors among asylum seekers were 'dissatisfaction regarding the length of the asylum procedure', 'uncertainty about residence status' and language difficulties. There was also a borderline significant difference between refugees and asylum seekers for levels of pre migration stressors (χ^2^= 3.96, df = 1, P = 0.05).

**Table 1:** Characteristics of study population

**Characteristic**	**Number (%) of Respondents**		
	
	**Refugees (n = 28)**	**Asylum Seekers (n = 60)**	**Statistics**
**Gender**			χ^2 ^= 0.13, df = 1, P = 0.72
	
Male	20 (71.4)	39 (65.0)	
	
Female	8 (28.6)	21 (35.0)	

**Mean Age in years (SD)**	36.2 (8.1)	32.8 (9.6)	t = -1.65, df = 86, P = 0.10
	
**Age Range (Years)**			χ^2 ^= 4.35, df = 3 P = 0.23
	
18–27	3 (10.7)	18 (30.0)	
	
28–37	13 (46.4)	24 (40.0)	
	
38–47	9 (32.1)	12 (20.0)	
≥ 48	3 (10.7)	6 (10.0)	

**Marital Status**			χ^2 ^= 4.39, df = 2 P = 0.11
	
Married	17 (60.7)	26 (43.3)	
	
Never been Married	8 (28.6)	31 (51.7)	
	
Divorced & Widowed	3 (10.7)	3 (5.0)	

**Education**			χ^2 ^= 8.68, df = 2 P = 0.01
	
Primary & None	4 (14.4)	28 (46.7)	
	
Secondary	10 (35.7)	14 (23.3)	
	
University	14 (50.0)	18 (30.0)	

**Pre migration Stressors**			χ^2 ^= 3.96, df = 1 P = 0.05
	
0–7 trauma events	22 (78.6)	34 (56.6)	
	
8+ trauma events	6 (21.4)	26 (43.3)	

**Post migration Stressors**			χ^2 ^= 19.74, df = 1 P < 0.01
	
Low level (<2.5)	21 (75.0)	15 (25.0)	
	
High Level (≥ 2.5)	7 (25.0)	45 (75.0)	

**Chronic Conditions**			χ^2 ^= 1.425, df = 1 P = 0.23
	
0–1	15 (53.6)	24 (40.0)	
	
>1	13 (46.4)	36 (60.0)	

**Mean Time in Ireland in months (SD)**	25.7 (14.5)	18.3 (13.6)	t = -2.32, df = 86 P = 0.02

### Univariable associations between residence status and general health, PTSD symptoms and anxiety/depression

In this analysis (Table 2), the association between residence status (refugees vs. asylum seekers) and poor general health failed to reach statistical significance (P > 0.05). However, asylum seekers had a significantly higher risk of PTSD and depression/anxiety symptoms (OR 6.3, 95% CI: 2.2–17.9; OR 5.8, 95% CI: 2.2–15.4 respectively).

**Table 2:** Univariable association between the characteristics of the study population and health outcomes

**Characteristic**	**Poor general health**		**PTSD symptoms**		**Depression/anxiety symptoms**	
	Number (%) of respondents with poor health	Unadjusted OR (95% CI)	Number (%) of respondents with symptoms	Unadjusted OR (95% CI)	Number (%) of respondents with symptoms	Unadjusted OR (95% CI)

**Residence Status **(Asylum seekers compared to refugees)						

Refugees	12 (42.9%)	2.0 (0.8–4.9) P = 0.14	6 (21.4%)	6.3 (2.2–17.9) P < 0.01	9 (32.1%)	5.8 (2.2–15.4) P < 0.01
					
Asylum seekers	36 (60.0%)		38 (63.3%)		44 (73.3%)	

**Post Migration Stressors **(mean score < 2.5 compared to ≥ 2.5)						

Mean Score < 2.5	15 (41.7%)	2.4 (1.0–5.8) P = 0.05	5 (13.9%)	18.6 (5.9–57.8) P < 0.01	12 (33.3%)	7.5 (2.9–19.5) P < 0.01
					
Mean Score = 2.5	33 (63.5%)		39 (75.0%)		41 (78.8%)	

**Pre Migration Stressors **(mean number '0–7' compared to > 8 past traumatic events)						

0–7	29 (51.8%)	1.4 (0.6–3.3) P = 0.49	23 (41.1%)	2.7 (1.1–6.8) P = 0.03	28 (50.0%)	3.6 (1.3–9.6) P = 0.01
					
8+	19 (59.4%)		21 (65.6%)		25 (78.1%)	

**Chronic Conditions **(mean number '0 or 1' compared to '>1' chronic conditions)						

0 or 1	18 (46.2%)	1.8 (0.8–4.3) P = 0.16	11 (28.2%)	5.3 (2.1–13.2) P < 0.01	16 (41.0%)	4.4 (1.8–11.0) P < 0.01
					
>1	30 (61.2%)		33 (67.3%)		37 (75.5%)	

OR Odds ratio, CI confidence interval, PTSD post traumatic stress disorder

Respondents with a high level of post migration stressors had a higher risk of poor general health status (OR 2.4, 95%CI: 1.0–5.8), PTSD (OR 18.6, 95% CI: 5.9–57.8) and depression/anxiety symptoms (OR 7.5 95% CI: 2.9–19.5). Pre migration stressors had no significant association with general health, but they had a significant positive association with the occurrence of PTSD symptoms (OR 2.7, 95% CI: 1.1–6.8) and depression/anxiety symptoms (OR 3.6, 95% CI: 1.3–9.6).

Respondents suffering from more than one chronic condition had an increased risk of PTSD (OR 5.3, 95% CI: 2.1–13.2) and depression/anxiety symptoms (OR 4.4, 95% CI 1.8–11.0), but no significant association was found with self reported poor general health.

Gender, length of time in Ireland and education level (Primary or less compared to secondary or above) were also explored in univariable analysis but had no significant effect on the outcomes for PTSD or depression/anxiety (data not shown).

### Multivariable associations between residence status and symptoms of PTSD and anxiety/depression

All variables that were significantly associated with PTSD and depression/anxiety from univariable analysis were included in multivariable logistic regression (Table 3). General health status was not explored in regression analysis as no significant association was found upon univariable analysis.

**Table 3:** Final regression model showing factors associated with PTSD and depression/anxiety symptoms

**Characteristics**	**PTSD Symptoms**		**Depression/Anxiety Symptoms**	
	
	**Adjusted OR(95%CI)**	**P Value**	**Adjusted OR(95%CI)**	**P Value**
Residence Status Refugees compared to Asylum Seekers	2.5 (0.6 – 10.0)^a^	0.19	3.0 (0.9 – 9.8)	0.08

Pre migration Stressors 0–7 compared to = 8	3.6 (1.1 – 11.9)^b^	0.04	3.3 (1.0 – 10.4)	0.04

Post migration Stressors <2.5 compared to = 2.5	17.3 (4.9 – 60.8)^b^	P < 0.01	3.9 (1.2 – 12.3)	0.02

Chronic Conditions 0–1 compared to >1	4.0 (1.3 – 12.7)^b^	0.02	3.4 (1.2–10.1)	0.03

^a ^is the adjusted OR for the model containing residence status. ^b ^is the adjusted OR after removal of residence status from the model.

For both regression models; residence status, post migration stressors, pre migration stressors and the presence of >1 chronic condition was entered into the model. In the PTSD model, the final regression model retained post migration stressors, pre migration stressors and chronic conditions, while the depression/anxiety regression model retained all three explanatory variables.

OR Odds ratio, CI confidence interval, PTSD post traumatic stress disorder

After adjusting for chronic conditions and pre and post migration stressors (Table 3), residence status was no longer significantly associated with symptoms of PTSD (OR 2.5, 95% CI: 0.6–10.0). After removal of residence status from the model, the association between pre migration stressors, post migration stressors and chronic conditions with PTSD remained. Residence status, pre migration stressors, post migration stressors and chronic conditions were retained in the regression model for depression/anxiety symptoms, however residence status was no longer a significant explanatory factor (OR 3.0, 95% CI: 0.9–9.8, Table 3).

Post migration stressors were the most significant risk factor for self reported PTSD and depression/anxiety symptoms (OR 17.3, 95% CI: 4.9–60.8; OR 3.9, 95% CI: 1.2–12.3 respectively). High levels of pre migration stressors and chronic conditions also had a significant positive association with the occurrence of these health outcomes. In contrast, residence status was not independently associated with the presence of PTSD or depression/anxiety symptoms, but appeared to act as a marker for other explanatory variables. Table 1 showed a strong relationship between residential status and levels of post migration stressors, and to a lesser degree, residential status and pre migration stressors, suggesting that residential status most likely acts as a marker for the presence of migration stressors.

### Utilisation of Health Care Services and use of Medication

Table 4 summarises and compares the use of health care services and medication between asylum seekers and refugees. The only statistically significant difference between asylum seekers and refugees in terms of health care services and medication use was in their use of general practice services (P = 0.03), with half of the refugees contacting their GP in last two months, compared to 73.3% of asylum seekers. No significant differences were found for hospital admissions, use of specialist services or the use of medications in the last 14 days.

**Table 4:** Utilisation of health services and medication use according to residence status

**Health Care Services (contact with service in previous period stated)**	**Refugees** **(n = 28)** **(%)**	**Asylum Seekers** **(n = 60)** **(%)**	**Unadjusted OR(95%CI)**	**P Value**
G.P. (2 months)	14 (50.0)	44 (73.3)	2.8 (1.1–7.0)	0.03

Hospitalisation (12 months)	4 (14.3)	12 (20.0)	1.5 (0.4–5.2)	0.52

Specialist (2 months)	6 (21.4)	17 (28.3)	1.5 (0.5–4.2)	0.49

Mental Health (12 months)	2 (7.1)	9 (15.0)	2.3 (0.5–11.4)	0.31

Dentist	8 (28.6)	13 (21.7)	0.7 (0.3–1.9)	0.48

**Medications (in last 14 days)**				

Any medication	17 (60.7)	43 (71.7)	1.6 (0.6–4.2)	0.31

Analgesics & antipyretics	13 (46.4)	32 (53.3)	1.3 (0.5–3.2)	0.55

Sedatives & sleeping tablets	3 (10.7)	11 (18.3)	1.8 (0.5–7.3)	0.37

Antidepressants	3 (10.7)	13 (21.7)	2.3 (0.6–8.9)	0.22

Other medications	11 (39.3)	31 (51.7)	1.7 (0.7–4.1)	0.28

## Discussion

### Principal findings

The results of the present study suggest that asylum seekers suffer from a significantly higher level of post migration stressors, have spent less time in Ireland and have a lower level of education compared to refugees (Table 1). In terms of health outcomes, asylum seekers appear to have a similar level of general health to refugees but they are more likely to suffer from symptoms of PTSD and depression/anxiety (Table 2). Higher levels of pre and post migration stressors and the presence of more than one chronic disease are independently associated with PTSD and anxiety/depression (Table 3). After adjusting for pre and post migration stressors and the presence of chronic conditions, residence status is no longer associated with PTSD or depression/anxiety, suggesting that residence status acts as a marker for other explanatory variables. As there is a strong relationship between residence status and post migration stressors (Table 1), it is likely that residence status acts as a marker for levels of post migration stressors.

In terms of health care utilisation, asylum seekers were found to use GP services more often than refugees. There was no statistically significant difference between these groups for use of dentists, medication, hospitalisation or mental health services.

### Context of other studies

The association between pre and post migration stressors and self reported mental health shown in this study (Table 2 and 3) supports the findings of other studies [13,16-20] and implies that mental health problems often develop and/or increase after arrival in Ireland. Factors such as social isolation, lack of work, cultural shock, language barriers, asylum procedure stress, fear of deportation and separation from children, have been suggested to be the major causes of post migration stress [16,17,20].

The association between residence status and mental health status (PTSD, depression/anxiety) in this study (Table 2) has also been shown in other countries [7,13], with asylum seekers having a higher level of self reported mental health problems. A long asylum procedure has also been associated with an increase in psychiatric disorders [21,22]. This association could be due to a number of factors including refugees feeling more a part of the community and more in control of their daily lives and subsequently suffering from lower levels of post migration stressors.

A review on the prevalence of mental disorders in refugees reported the prevalence of PTSD ranging from 2% in Vietnamese/Chinese refugees to 45% in Cambodian refugees [1], in this study the prevalence was 21.4% (Table 2). The high variability in prevalence rates may be due to differences between countries in their treatment of refugees or it could be due to the use of different tools to assess the presence of PTSD and depression/anxiety symptoms in the different studies. The prevalence was relatively high in this study compared to studies carried out in other European countries [2,7,13]. A recent study from Ireland reported that asylum seekers were five times more likely to be diagnosed with psychiatric illness than Irish Citizens [23].

Considering the higher level of self reported mental health problems among asylum seekers, it might be expected that this group would utilise mental health services more often. However, that was not the case in this study and health service utilisation only differed between refugees and asylum seekers in the use of GP services (Table 4), with asylum seekers utilising GP services more than refugees. A recent study from the Netherlands [24], reported on health service use of Iraqi asylum seekers and found the same unmet health need, with a high percentage of those with psychiatric disorders not utilising mental health services. Another study from the Netherlands on refugees and asylum seekers reported no differences in these two groups in terms of self reported use of health care services. They also reported that both these groups may experience problems with accessing mental health care due to cultural and/or language barriers [25].

Overall it is still unclear if asylum seekers are not being offered adequate mental health services (due to lack of availability or communication problems) or if they are unwilling to use them for cultural or personal reasons. What is clear is that there is an issue of unmet health need within this population that ought to be clarified and addressed. A recent study from the UK on refugees' mental health needs suggested that refugees have a relatively high level of mental health services need but a low level of service utilisation, with GP services being the most utilized service [26].

### Shortcomings of present study

The size of our study was relatively small and confined to a geographic area in the North West of Ireland. This region of Ireland has a relatively small population compared to the capital city, Dublin (90,000 compared to 1.2 million in 2006 [27]) and would not be ethnically diverse. It is therefore possible that refugees or asylum seekers in this region undergo a different level or type of post migration stress compared to those who remain in a large city such as Dublin. However, there is a lack of studies comparing the health status of asylum seekers and refugees especially in the Irish context. Other explanatory variables could have been included in our analysis. For example unemployment is known to have a detrimental effect on a persons mental health [28], and therefore this could have been explored in the refugee group.

One of the strengths of this study is the use of validated instruments, which have been developed or adapted for cross cultural studies [5,6]. We chose cut-off scores for these instruments that are commonly used so our results would be directly comparable to other studies. However, we acknowledge that the instruments and the cut-points used have not been validated in our population, which was community based and contained a wide range of nationalities.

### Future studies

Our study suggests that more research is needed on the effect of the asylum process on mental health and that interventions are needed that reduce post migration stressors or introduce coping strategies.

The lack of utilisation of mental health services should be further investigated to discover if patients should be referred to these services or if immigrants are satisfied that their mental health problems are being adequately addressed by GPs.

### Health policy recommendations

The provision of interpreters and training of health service providers to promote cultural awareness, particularly within the mental health service and GP settings would facilitate the utilisation of these services by refugees and asylum seekers. While education programmes within direct provision centres on Irish culture, available health services and life skills required for the new environment would also help in narrow the gap in communication and cultural differences.

Our results suggest that living within direct provision centres adversely affects mental health; it isolates asylum seekers from the local community, and limits their integration and understanding of the society they wish to join. In addition, preventing asylum seekers from working is known to lead to mental health problems and issues of self esteem [28].

## Conclusion

Asylum seekers have a higher level of self reported PTSD and depression/anxiety symptoms compared to refugees. However, residence status appears to act as a marker for post migration stressors. Compared to refugees, asylum seekers utilise GP services more often. Despite the higher level of mental health problems in asylum seekers, no difference was found between these groups in the utilisation of mental health services.

The findings of this study illustrate the need for preventative measures or coping strategies in addition to psychological and psychiatric treatment. Addressing pre migration traumas with psychological support, should be combined with a strategy to increase coping or reduce post migration stressors, as they are a strong predictor of mental health problems in these vulnerable groups.

## Competing interests

The authors declare that they have no competing interests.

## Authors' contributions

MT and TF were involved in the design of the study. MT carried out the fieldwork for the study, performed the statistical analysis and drafted the manuscript. KOB was involved in the statistical analysis and along with MT and TF redrafted the manuscript. All authors read and approved the final manuscript.

## Pre-publication history

The pre-publication history for this paper can be accessed here:


